# Mechanical Performance Evaluation of Negative-Poisson’s-Ratio Honeycomb Helmets in Craniocerebral Injury Protection

**DOI:** 10.3390/ma18102188

**Published:** 2025-05-09

**Authors:** Bin Yang, Xingyu Zhang, Yang Zheng, Peng Zhang, Xin Li, Jinguo Wu, Feng Gao, Jiajia Zou, Xuan Ma, Hao Feng, Li Li, Xinyu Wei

**Affiliations:** 1 School of Transportation Engineering, Nanjing Institute of Technology, Nanjing 211167, China; yangb123@126.com (B.Y.); y00450240142@njit.edu.cn (P.Z.); wujg8848@163.com (J.W.); g1850696719@163.com (F.G.); 18105178207@163.com (J.Z.); 15252359272@163.com (X.M.); y00450240411@njit.edu.cn (H.F.); y00450240321@njit.edu.cn (L.L.); y00450240338@njit.edu.cn (X.W.); 2 School of Automotive and Transportation Engineering, Jiangsu University, Zhenjiang 212013, China; 15250963612@163.com

**Keywords:** negative-Poisson’s-ratio structure, re-entrant hexagonal honeycomb, helmet liner, craniocerebral injury protection, biomechanics

## Abstract

Helmets are crucial for protecting motorcycle riders from head injuries in accidents. This study proposes a helmet pad design based on a negative-Poisson’s-ratio (NPR) structure and comprehensively evaluates its protective effect on head injuries. A concave hexagonal honeycomb structure was embedded into the energy-absorbing lining of a motorcycle helmet, and finite element collision simulations were conducted according to the ECE R22.05 standard. These simulations compared and analyzed the differences in protective performance between concave hexagonal honeycomb helmets with different parameter configurations and traditional expanded polystyrene (EPS) helmets under flat anvil impact scenarios. Using biomechanical parameters, including peak linear acceleration (PLA), head injury criterion (HIC), intracranial pressure (ICP), maximum principal strain (MPS), and the probability of AIS2+ traumatic brain injury, the protective effect of the helmets on traumatic brain injury was evaluated. The results showed that when the wall angle of the honeycomb structure was 60°, honeycomb helmets with wall thicknesses of 0.8 mm and 1.0 mm significantly reduced PLA and HIC values. In particular, the honeycomb helmet with a wall thickness of 1.0 mm reduced ICP by 25.7%, while the honeycomb helmet with a wall thickness of 1.2 mm exhibited the lowest maximum principal strain in the skull compared to EPS helmets and reduced the probability of AIS2+ brain injury by 7.2%. Concave hexagonal honeycomb helmets demonstrated an excellent protective performance in reducing the risk of traumatic brain injury. These findings provide important theoretical foundations and engineering references for the design and optimization of new protective helmets.

## 1. Introduction

The development of transportation infrastructure and the growing intricacy of urban roadway systems have prompted heightened public awareness of cyclist safety. Statistical evidence from the National Bureau of Statistics of China [1] indicates a statistically significant rise in bicycle- and motorcycle-related traffic accidents, with the rate of such incidents increasing from 19.3 to 19.8 percent between 2019 and 2023. This upward trajectory underscores cyclist safety as a salient societal issue demanding urgent intervention. Within this scenario, helmet engineering arises as a vital protective strategy, where structural design and material properties fundamentally influence the capacity to reduce traumatic brain injuries.

Traditional helmet configurations primarily rely on stiff outer shells and energy-absorbing padding layers. Nevertheless, recent research has noted a rise in novel design concepts that seek structural advancements beyond conventional layouts. Of particular interest is the auxetic (negative Poisson’s ratio, NPR) structural helmet. This groundbreaking design demonstrates exceptional energy dissipation capabilities. The negative Poisson’s ratio characteristic, which causes lateral expansion when subjected to axial tension, contrasts with the behavior of conventional materials, which exhibit a positive Poisson’s ratio. As a result, these metamaterials possess superior impact mitigation properties. Recent advancements have enabled the application of NPR structures across various engineering sectors, including in aerospace systems, automotive safety components, and personal protective equipment. Within the personal protective equipment domain, adopting NPR-based design principles has revolutionized helmet protection technology. This innovation has significantly enhanced energy absorption performance during impact scenarios. For instance, Li et al. [2] developed a crossed star-shaped honeycomb (CSSH) derived from the traditional star-shaped honeycomb (SSH). By converting the horizontal and vertical walls into crossed sloping walls, this design improved energy absorption capacity through horizontal adjustments and deformation stability through vertical adjustments. Compression testing indicated that CSSH performs better than SSH. Its specific energy absorption (SEA) is 245% higher than that of SSH.

Moreover, Lin et al. [3] introduced a self-similar concentric star honeycomb (SCSH) and investigated its dynamic impact behavior at three different impact velocities. Their findings suggested that SCSH showed higher plateau stress and better energy absorption than SSH at different velocities, especially at low and medium speeds. Song et al. [4] investigated the buffering performance of sinusoidal negative-Poisson’s-ratio (NPR) honeycomb structures under varying impact velocities using numerical simulations, experimental verification, and multi-objective optimization design. Their research revealed that the wall thickness gradient sinusoidal NPR honeycomb structure (WTG-CM) demonstrated the lowest initial peak crushing force (PCF) and the highest specific energy absorption (SEA) under low-speed impacts. In contrast, the amplitude gradient sinusoidal NPR honeycomb structure (AG-CM) exhibited superior buffering and energy absorption characteristics under medium- to high-speed impacts. Qin et al. [5] introduced two axisymmetric four-chiral honeycomb structures (ATCH-1 and ATCH-2) derived from the traditional four-chiral honeycomb structure (TCH). Through finite element modeling, they analyzed the influence of various parameters on the deformation modes, energy absorption characteristics, and NPR effects of these structures. The results indicated that ATCH-1 and ATCH-2 achieved a higher SEA and a more pronounced NPR effect compared to TCH at an impact velocity of 10 m/s.

Furthermore, Zhu et al. [6] introduced a novel elliptical annular re-entrant honeycomb (EARE) by incorporating an elliptical annular structure into the unit cell of a traditional re-entrant honeycomb (RE). This alteration not only permitted lateral deformation, but also provided supplementary longitudinal support without obstruction. Quasi-static compression tests demonstrated that, compared to a conventional RE honeycomb with the same wall thickness, the EARE not only enhanced the negative Poisson’s ratio (NPR) effect, with the Poisson’s ratio decreasing by 5.19%, but also elevated plateau stress and specific energy absorption (SEA) by 171.63% and 28.03%, respectively. These enhancements can considerably improve the stiffness and energy absorption capabilities of traditional honeycomb structures. In a separate study, Yu et al. [7] fabricated assisted re-entrant honeycomb sandwich structures utilizing carbon/epoxy prepregs, followed by an investigation into their compressive properties and damage behavior under quasi-static compression. They evaluated the following three distinct gradient configurations of assisted re-entrant honeycomb structures: average, unidirectional, and bidirectional. The results of the compression experiments indicated that the average honeycomb structure achieved an optimal energy absorption performance, while the bidirectional honeycomb structure excelled in terms of NPR characteristics. Lastly, Yang et al. [8] designed four different negative-Poisson’s-ratio structures with varying cellular forms—concave triangular, concave hexagonal, honeycomb hexagonal, and chiral structures—which were manufactured via 3D printing. Their analysis of mechanical and impact properties suggested that among the tested designs, the concave hexagonal structure demonstrated the best energy absorption performance. Hai et al. [9] introduced an enhanced dual-honeycomb structure (MDRH) and investigated its deformation modes, nominal stress–strain curves, and specific energy absorption (SEA) under varying impact velocities. They compared the performance of MDRH with traditional concave honeycomb (TRH) materials. The results demonstrated that the MDRH structure significantly extended the range of effective modulus and negative Poisson’s ratio (NPR) values. Across different impact velocities, MDRH consistently exhibited high platform stress and a superior specific energy absorption, showcasing its exceptional impact resistance.

Although existing research has validated the advantages of negative-Poisson’s-ratio (NPR) structures in energy absorption and dynamic response, their practical applications still face significant limitations. Currently, most studies focus on the mechanical performance testing of individual structures [10,11,12], lacking a systematic analysis of the coupling effects between NPR structures and head biomechanics. This gap results in an unclear adaptability of these structures in real-world helmet protection scenarios. To address this, this study integrated NPR structural design into helmet padding. We developed a finite element model of the helmet, incorporating an NPR pad, and paired it with a detailed human head model. Drop test simulations were conducted in accordance with the European ECE R22.05 standard [13] to evaluate the model’s effectiveness. Additionally, the impact resistance of the NPR helmet with various pad parameter configurations was compared and analyzed against traditional expanded polystyrene foam (EPS) helmets. A systematic analysis was performed to assess the influences of wall thickness and wall angle on key cranial injury indicators, including head injury criteria (HIC), intracranial pressure (ICP), peak linear acceleration (PLA), and maximum principal strain (MPS). This analysis enabled the determination of an optimal parameter combination, providing a theoretical foundation and engineering reference for the design and optimization of advanced protective helmets. Furthermore, this study elucidates the dynamic response mechanisms of NPR structures in helmet design, offers insights for optimizing energy absorption pathways, and advances helmet protection technology. Ultimately, these contributions aim to enhance the safety of motorcycle riders during accidents and reduce the risk of cranial injuries.

## 2. Models and Methods

### 2.1. Finite Element Model of the Head

#### 2.1.1. Model Construction

**Figure 1:**
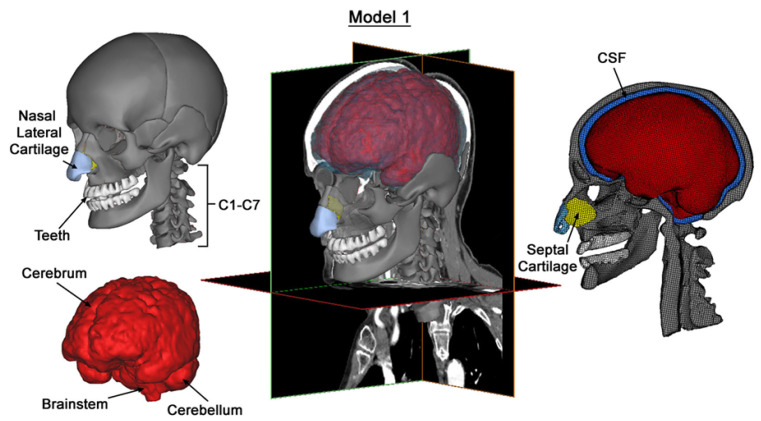
Three-dimensional geometric model of the head [24].

The anatomical specifics of the human skull and brain were obtained from a comprehensive set of 460 axial CT images, each with an in-plane resolution of 512 × 512 pixels. These images, which had a pixel size of 0.488 mm and a slice thickness of 1.0 mm, were acquired from a contrast-enhanced CT scan dataset of a middle-aged individual. The dataset was obtained from the medical records of the National University Health System (NUHS). Subsequently, the CT images were processed in Mimics v14.0 (Materialise, Leuven, Belgium) software for image filtering, segmentation, and boundary-contour extraction, and a 3D head model was successfully reconstructed. Magnetic resonance imaging (MRI) was used to automatically outline the white matter, grey matter, and ventricular structures of the brain. Ultimately, the human brain was split into several components, as follows: the cranium (comprising the ethmoid sinus, frontal bone, maxilla, mandible, nasal bone, occipital bone, pterygoid process of the sphenoid bone, parietal bone, temporal bone, and zygomatic bone); the cranial brain (comprising white matter, grey matter, cerebellum, lateral ventricles, third ventricle, fourth ventricle, midbrain, cerebral pons, medulla oblongata, and spinal cord); cerebrospinal fluid (CSF); soft tissues (comprising the scalp, adipose tissues, muscular tissues, inferior turbinate, middle turbinate, and superior turbinate); and air sinuses. In the field of brain injury mechanics, Zhou et al. [14] enhanced the original cerebrospinal fluid (CSF) modeling approach by transitioning to a fluid–structure interaction (FSI) method. This modified model demonstrated an improved validation performance in terms of brain–skull relative motion and provided more accurate predictions of acute subdural hematoma (ASDH). Similarly, Willinger et al. [15] employed the arbitrary Lagrangian–Eulerian (ALE) formulation to simulate FSI, concluding that the pressure response in this case aligned more closely with experimental data compared to solid interface modeling. These studies highlight that the FSI method offers a more comprehensive assessment of head biomechanical behavior, including the pressure distribution and interaction of CSF with brain tissue. However, in this study, we opted to model CSF as a solid rather than employing FSI. This decision was driven by the primary objective of evaluating the energy absorption performance and protective efficacy of negative-Poisson’s-ratio (NPR) helmet pads under impact loads, rather than analyzing the detailed fluid dynamics of CSF. By simplifying the model, we were able to focus on investigating the influences of the geometric design and material properties of NPR structures on head protection performance within a reasonable computational time and resource framework. Furthermore, through comparisons with experimental data, our model demonstrated a strong accuracy and reliability in predicting intracranial pressure (ICP) and brain tissue strain. The geometric model is presented in Figure 1, and the material parameters of each component are provided in Table 1.

**Figure 2:**
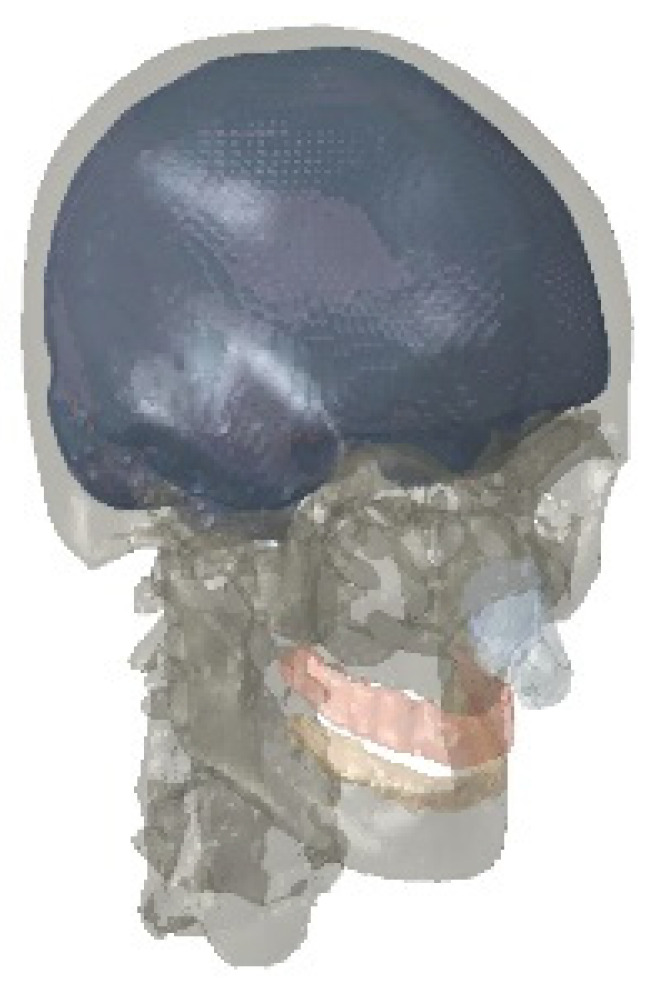
Finite element model of the head.

After the model construction was completed, Steiner points were organized into a continuous hierarchy. The enclosed surface areas were populated with a tetrahedral mesh using the Delaunay triangulation method in HyperMesh v11.0 (Altair HyperWorks, Troy, MI, USA). For the tetrahedral mesh, the Laplace smoothing technique was applied to perform node insertion and deletion operations, and local repartitioning was carried out to complete the mesh optimization process. The finalized finite element model consisted of 327,536 nodes and 1,173,039 linear tetrahedral elements, featuring an average edge length of roughly 1.57 mm and an aspect ratio of 1.61. The finite element model is illustrated in Figure 2 [16].

Based on the established finite element model [17], cerebrospinal fluid (CSF), the human skull, and other skeletal tissues, including cartilage and teeth, are considered as linear-elastic materials, whereas brain tissue is regarded as viscoelastic.

Equation (1) characterizes the shear properties of the linear viscoelastic behavior of the brain tissue.

(1) $$G(t)=G_{\infty }+(G_{0}-G_{\infty })e^{-\beta t}$$

where $G_{\infty }$ is the long-term shear modulus (MPa), $G_{0}$ is the short-term shear modulus (MPa), and β is the decay factor in s^−1^.

**Table 1:** Head model material parameters.

	Material Type	Element Type	Young’s Modulus *E* (MPa) *G*(*t*) = *G_∞_* + (*G*_0_ − *G*_∞_) *e^−^^βt^*	Poisson’s Ratio	Density (kg m^−3^)	References
Brainstem	Linear viscoelastic	Tetrahedral	*G*_0_ = 0.528, *G*_∞_ = 0.168, *β* = 35 s^−1^	0.48	1140	[17,18,19,20,21,22,23,24]
Cerebral peduncle	Linear viscoelastic	Tetrahedral	*G*_0_ = 0.0225, *G*_∞_ = 0.0045, *β* = 80 s^−1^	0.4996	1060	[25]
Cerebellum	Linear viscoelastic	Tetrahedral	*G*_0_ = 0.528, *G*_∞_ = 0.168, *β* = 35 s^−1^	0.48	1140	[17,18,19,20,21,22,23,24]
CSF	Linear elastic	Tetrahedral	*E* = 1.314	0.4999	1040	[23]
Gray matter	Linear viscoelastic	Tetrahedral	*G*_0_ = 0.528, *G*_∞_ = 0.168, *β* = 35 s^−1^	0.4996	1040	[24,25]
Lateral cartilage	Linear elastic	Tetrahedral	*E* = 30	0.45	1500	[26]
Septum cartilage	Linear elastic	Tetrahedral	*E* = 9	0.32	1500	[27]
Bone	Linear elastic	Tetrahedral	*E* = 8000	0.22	4740	[24]
Soft tissues	Linear elastic	Tetrahedral	*E* = 16.67	0.46	1040	[24,28]
Tooth	Linear elastic	Tetrahedral	*E* = 2070	0.3	2250	[29,30]
Ventricles	Linear elastic	Tetrahedral	*E* = 1.314	0.4999	1040	[23]
White matter	Linear viscoelastic	Tetrahedral	*G*_0_ = 0.041, *G*_∞_ = 0.0078, *β* = 700 s^−1^	0.4996	1040	[25]

#### 2.1.2. Experimental Validation of Nahum’s Intracranial Pressure

In the 1970s, Nahum et al. [31] carried out a crucial investigation into linear impacts using human cadavers, with a specific emphasis on frontal impacts on seated subjects. During the experiment, a rigid cylindrical impactor weighing 5.59 kg, moving at a velocity of 9.94 m s^−1^, struck the cadaver along the midsagittal plane. Post-collision, the head was rotated 45 degrees from the anatomical plane and tilted forward. This setup allowed for a thorough analysis of cranial movements.

**Figure 3:**
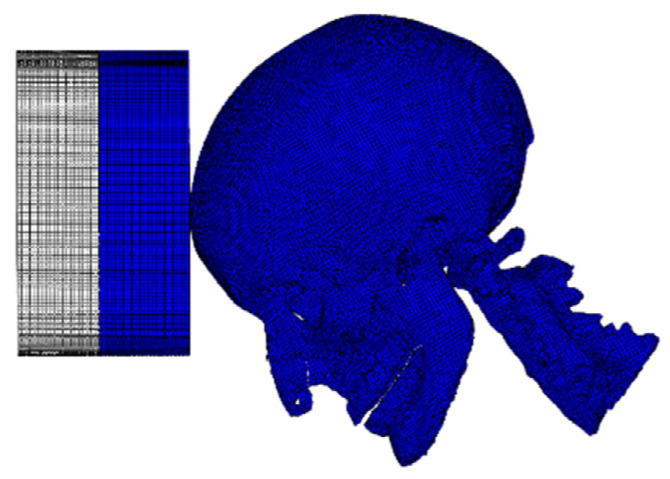
The setting conditions of simulation.

Although Nahum et al. [31] performed multiple cadaveric experiments, this study concentrated on Experiment 37, as described in their paper. Our objective was to verify the accuracy of the head model in predicting intracranial pressure using Abaqus v6.14 (SIMULIA, Rhode Island, RI, USA), utilizing the extensive data and associated curves provided in their work. The Frankfurt anatomical plane of the model was positioned at a 45° angle relative to a rigid cylindrical impactor with a diameter of 152.4 mm and a thickness of 40 mm. Additionally, it was aligned with the superior margins of the orbital rim and auditory canals to mirror the experimental conditions. The impactor’s velocity was directed through the center of mass of the head model to ensure only translational motion and eliminate any rotational component. Figure 3 depicts the simulated collision that occurred in the frontal region at a velocity of 9.94 m s^−1^.

**Figure 4:**
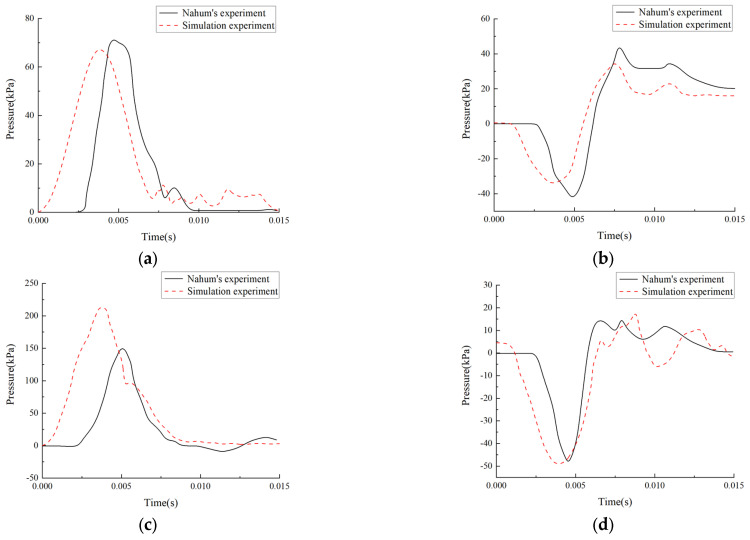
The comparison of time course of intracranial pressure between simulation and cadaver experiment: (**a**) parietal; (**b**) occipital; (**c**) impact side; and (**d**) impact opposite side.

Drawn from the parameters of Experiment 37, as reported by Nahum et al., the simulation collision duration was designated as 15 ms. In line with the experimental setup, upon simulation completion, time series data on intracranial pressure in the parietal, occipital, direct impact, and contralateral impact regions were gathered. As depicted in Figure 4, these gathered data were subsequently compared and analyzed alongside the corresponding curves obtained from Nahum’s Experiment 37.

A strong correlation was found between the head model developed herein and the data observed at the four critical locations. Despite a notable divergence between the simulation outcomes and the actual data in terms of peak intracranial pressure (ICP), the model reliably predicted the temporal pressure variations. Specifically, the measured intracranial pressure (ICP) was 151 kPa at 5.0 ms, whereas the simulation foretold a peak value of 214 kPa at 3.7 ms on the impact side. This divergence exemplifies the differing responses of the model and the physical cadaver. Notwithstanding the discrepancies in the model’s dimensions and tissue characteristics, the maximum pressures at the remaining three sites closely paralleled the observed data. These differences do not diminish the head model’s utility. Even with inconsistencies between the simulation results and cadaver experiments, the model’s validity was largely affirmed.

#### 2.1.3. Experimental Validation of Trosseille’s Intracranial Kinetic Response

In 1992, Trosseille et al. [32] executed a groundbreaking experiment on adult cadaver head impacts to verify the finite element model of the head. To replicate the collision between a human head and a vehicle part during an accident, they performed six impact tests on the heads of three recently acquired, unembalmed cadavers. The cadavers were positioned in a seated posture and a 23.4 kg impactor was utilized in the tests. The impactor’s size and velocity varied across the tests, targeting different head areas, such as the face, forehead, and temples. Pressure readings were collected at corresponding sites on the skull. Importantly, the impact force’s line of action was not aligned with the head’s center of mass, resulting in both translational and rotational head accelerations. Table 2 outlines the detailed experimental conditions for each of the three cadaver specimens.

**Figure 5:**
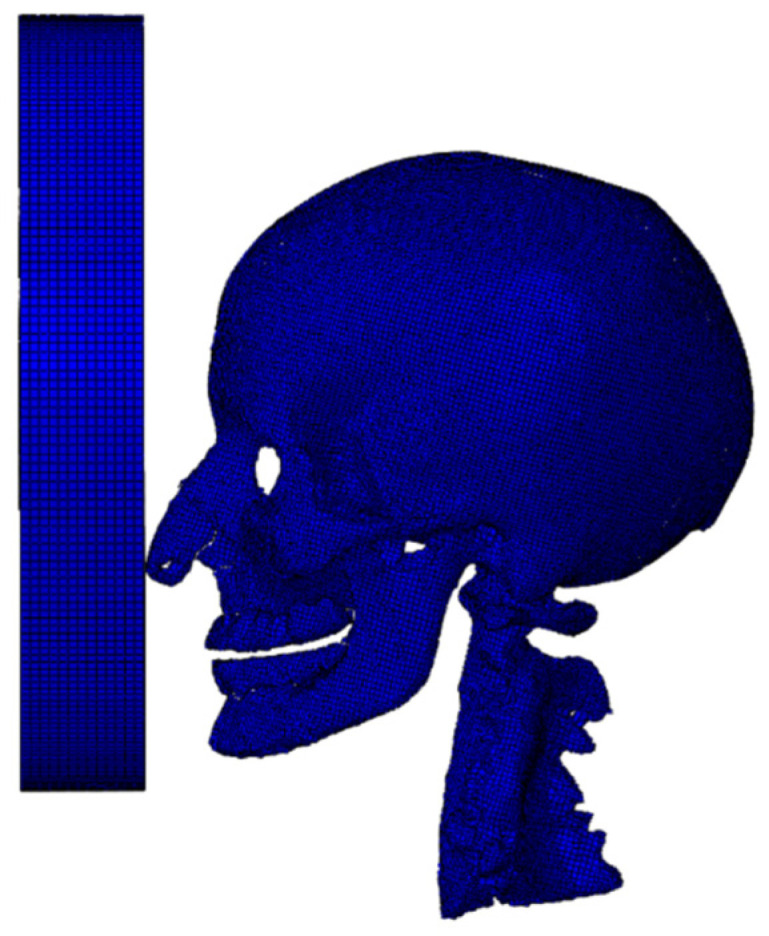
Trosseille corpse model collision experiment.

**Figure 6:**
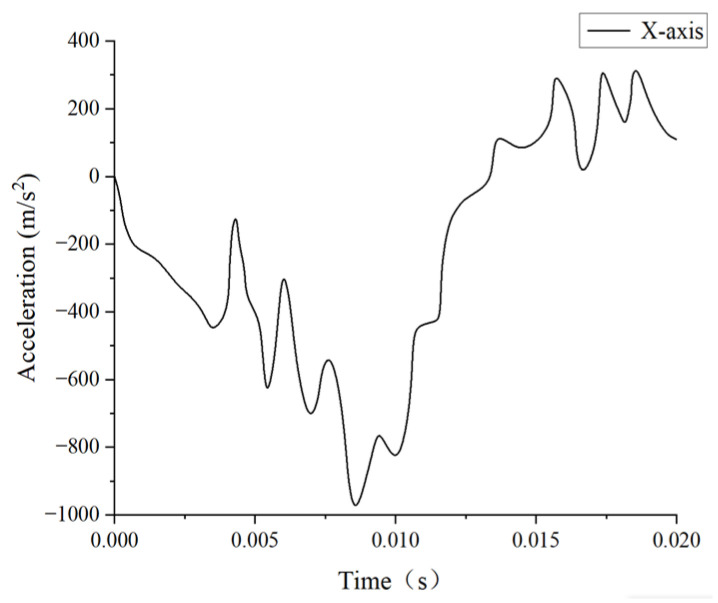
*X*-axis translation acceleration–time curve.

**Figure 7:**
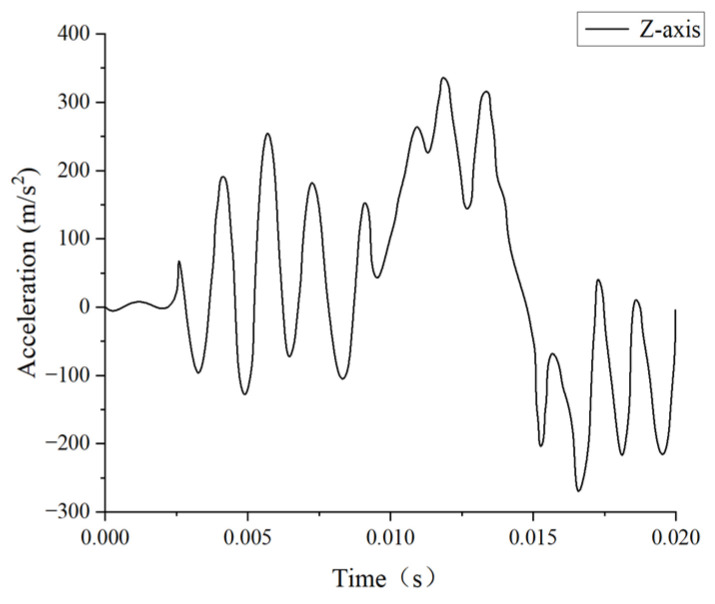
*Z*-axis translation acceleration–time curve.

**Figure 8:**
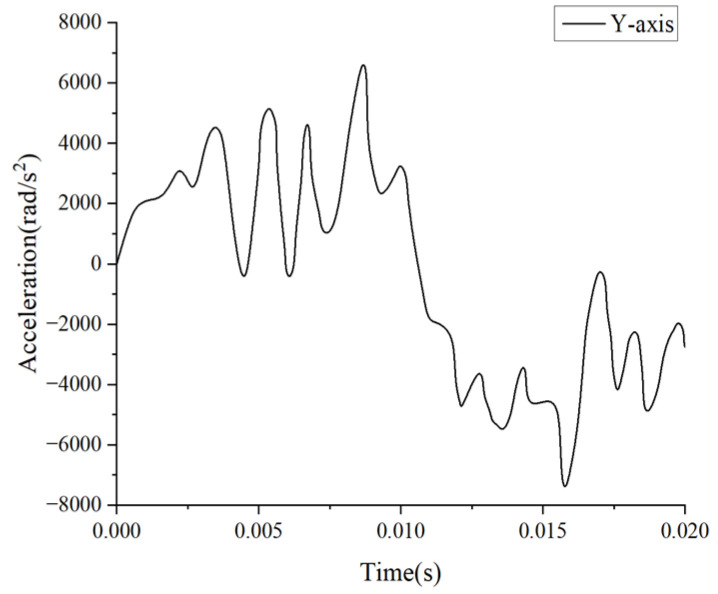
*Y*-axis translation acceleration–time curve.

In this study, we selected experiment MS428-2 as a comparative validation test to replicate the conditions reported by Trosseille et al. [32] and recreate the scenario of an automobile accident collision. To achieve this, we replaced the steering wheel with a rigid cylindrical object with a diameter of 250 mm, a height of 40 mm, and an inertial mass of 23.4 kg. This object was then used to impact the face of the head finite element model, as shown in Figure 5. In Trosseille’s experiment, the cadaver was placed in a seated position with the head connected to the body, with the impact lasting up to 15 ms. As a result, the influence of structures like the neck on the head’s kinematic response could not be ignored, unlike in Nahum’s experiment. Therefore, we imposed fixed boundary conditions on the lower-surface nodes of the C7 vertebrae in our simulation. Additionally, since the collision occurred in the head’s midsagittal plane, we defined the skull as a rigid body and disregarded the translational acceleration along the *Y*-axis. The initial boundary conditions for the simulation were derived from Trosseille’s cadaver experiments, specifically the translational accelerations along the *X*- and *Z*-axes of the center of mass of the head, as well as the rotational acceleration about the *Y*-axis. These conditions were applied to the superior part of the rigid skull, as illustrated in Figure 6, Figure 7 and Figure 8.

**Figure 9:**
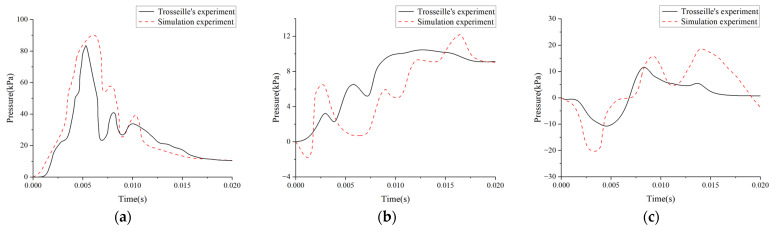
Comparison of model simulated craniocerebral pressure and experimental pressure data curves: (**a**) frontal lobe pressure–time curve; (**b**) temporal lobe pressure–time curve; and (**c**) occipital lobe pressure–time curve.

A 20 ms simulation of the intracranial kinetic response, based on Trosseille’s experiment, was performed. After the simulation, pressure data from the frontal, temporal, and occipital lobes were gathered and contrasted with Trosseille’s experimental data. The comparison results are presented in Figure 9.

Figure 9 presents the simulated pressure curves for the frontal, temporal, and occipital regions alongside the experimental data. Overall, the simulated pressure curves exhibit closer agreement with the experimental findings, and the pressure distribution trends demonstrate strong correspondence across all three regions. Regarding peak pressure, the frontal region’s value lies within 10% of the experimental value, indicating a high degree of accuracy. However, the simulated pressure values in the occipital and temporal regions are marginally higher than the experimental values. In terms of curve shape, the frontal and occipital regions align well with the experimental data. By contrast, the simulated temporal pressure initially becomes negative, whereas the experimental curve approaches zero. Additionally, the timing and duration of peak pressure show minor differences from the experimental results.

Such errors have been documented in previous studies [33,34]. Based on prior research, the potential causes can be attributed to the following two primary factors: (1) there is a significant difference between the model and the cadaveric specimens used in experiments. In the model, the cerebrospinal fluid (CSF) is depicted as a solid element with a low shear modulus, which does not accurately represent the pressure distribution and solid–liquid interface interactions. (2) In experimental settings, the impactor generates contact forces that deform the skull and transfer forces to the intracranial area. In contrast, the simulation employs direct acceleration loading, omitting neck effects that may influence the results. In conclusion, although some discrepancies between the simulation and experimental data are unavoidable, the overall agreement remains acceptable.

#### 2.1.4. Experimental Validation of Hardy’s Craniocerebral Relative Displacement

**Figure 10:**
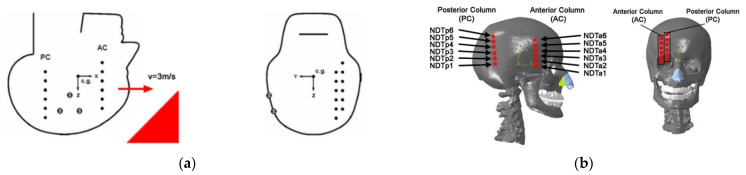
Comparison of head marker positions: (**a**) Hardy’s cadaver experiment [35] and (**b**) simulation experiment [23].

**Figure 11:**
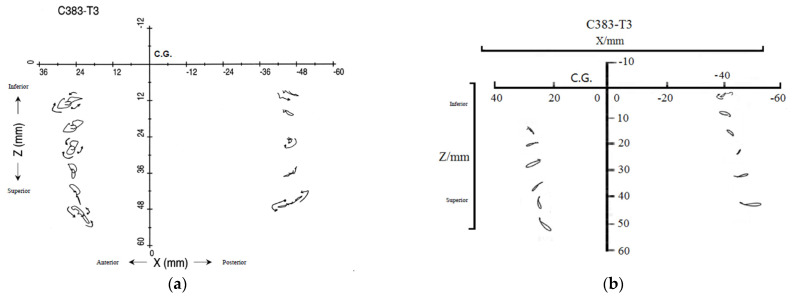
The tracks of NDTs: (**a**) Hardy’s cadaver experiment and (**b**) simulation experiment.

In 2001, Hardy et al. [35] performed cadaveric head impact research utilizing a high-speed biplane X-ray system and neutral density targets (NDTs) designed to closely mimic brain density. The primary objective of this research was to leverage advanced imaging technology to observe the trajectories of brain regions interacting with the skull. In this experiment, the researchers divided 12 NDTs into two groups, placing 6 in each of the temporoparietal and occipitoparietal regions of cadaveric skulls. The skulls were then impacted in the occipital region by a rigid impactor at velocities ranging from 2 to 4 m s^−1^. The high-speed X-ray system recorded the movement of head markers during impact, while a specialized sensor measured the relative displacement between cranial and cerebral tissues. This setup allowed for precise observations of kinematic characteristics at low velocities. Figure 10a shows the locations of the NDTs in Hardy’s research within the required coordinate system. Due to the minimal displacement of the NDTs along the *Y*-axis, Figure 11a illustrates their trajectories in the *XZ* plane during impact.

**Figure 12:**
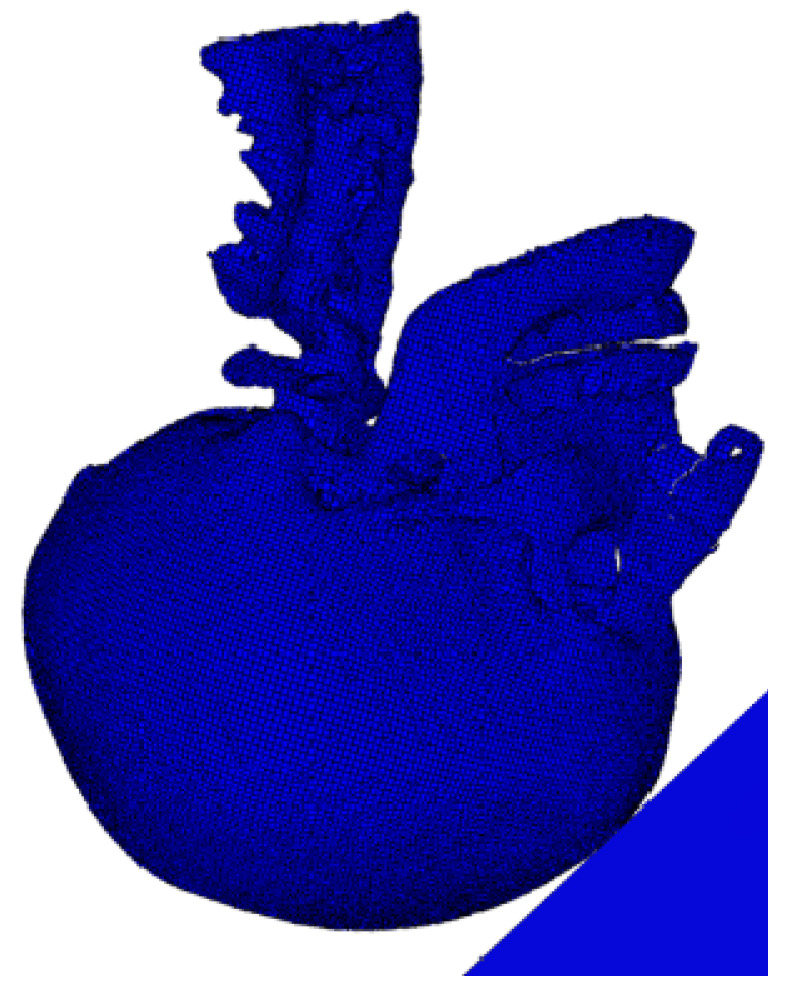
The schematic diagram of impact direction and position in collision simulation.

In the C383-T1 experiment, the head model’s accuracy was verified by analyzing the relative displacement data between the skull and brain tissue. Simulation tests were conducted using Abaqus software. As shown in Figure 12, the head model underwent impact simulations on an inclined plane. The model’s material properties included a density of 1185 kg m^−3^, a Poisson’s ratio of 0.4, and a Young’s modulus of 3.2 GPa. To replicate Hardy’s experimental conditions, the inclined-plane simulation used the parallel sides of the model as boundary conditions and restricted the bottom surface of the neck to five degrees of freedom. Additionally, as depicted in Figure 10b, two marker columns were strategically placed in the right cerebral hemisphere. The anterior marker (NDTa) was positioned in the temporoparietal region, while the posterior marker (NDTp) was located in the occipitoparietal region.

Figure 11b illustrates the motion paths of the NDTs observed in the simulation. The impactor was aligned with the midsagittal plane, resulting in minimal displacement along the *Y*-axis during the 60 ms dynamic response simulation. Post-impact, the simulation indicated that nearly all NDTs moved in unison with the brain tissue. However, the brain tissue’s movement relative to the skull displayed a noticeable lag, along with backward and downward displacements that peaked before recovering. The brain tissue and skull followed a circumferential path, with displacement magnitude varying by location. The simulation suggested that the maximum NDT displacement occurred within approximately 3 to 5 ms, aligning with the 4 to 6 ms range observed in Hardy’s experiment. This consistency implies a fundamental agreement in displacement trends.

Nonetheless, the NDT trajectory patterns in the simulation were more linear, differing from the figure-of-eight patterns seen in Hardy’s experiments. This suggests that the finite element model represented more uniform intracranial motion. Additionally, the displacement values of NDTa1, NDTp3, and NDTp6 in both directions were lower than those in Hardy’s experiment but remained within comparable ranges. The misalignment between the head model’s center of mass and that of the actual cadaver, due to dimensional differences between the model and experimental specimens, also contributed to reduced NDTa3 displacement along the *Z*-axis. Moreover, the simulation employed a viscoelastic model treating brain tissue as homogeneous, without distinguishing between gray and white matter. This contrasts with the actual brain tissue’s complex, heterogeneous nature, impacting the simulation accuracy.

In this study, a simplified brain model was employed, treating brain tissue as a uniform linear viscoelastic material and modeling cerebrospinal fluid (CSF) as a solid to reduce computational complexity. While this model demonstrated a good accuracy and reliability in simulating head impact responses and was validated against experimental data, it does have certain limitations.

First, brain tissue exhibits anisotropic and nonlinear mechanical behavior, with properties varying across different regions and under different degrees of deformation—characteristics that our model does not capture. Second, modeling CSF as a solid overlooks its fluid dynamics behavior, which may affect the accurate simulation of interactions between brain tissue and the skull, particularly in terms of key biomechanical parameters such as CSF pressure distribution and brain tissue displacement. Additionally, this study did not account for the influence of the neck on head movement. In real-world accident scenarios, the flexibility and muscle tension of the neck significantly affect the trajectory and acceleration of head motion.

Nevertheless, given that the primary objective of this study is to evaluate the impact of helmet design on the risk of traumatic brain injury (TBI), the current simplifications remain within an acceptable range. This approach provides a valuable theoretical foundation and engineering reference for the design and optimization of helmets.

While there were some differences between this study’s simulation results and Hardy’s experimental data, the deviations in the predicted displacements and overall curve profiles remained within an acceptable range when compared with the empirical findings. Overall, this paper validates the head finite element model by comparing it with Nahum’s intracranial pressure tests, Trosseille’s intracranial kinetic response research, and Hardy’s craniocerebral relative displacement experiments. Based on these investigations, the biomechanical properties of the model, including intracranial pressure and relative craniocerebral displacement, were thoroughly examined and compared with the experimental data. The experimental findings indicated that the developed head finite element model is both stable and biologically plausible, accurately replicating the biomechanical impacts of traffic collisions. Therefore, the model is highly suitable for practical applications, such as evaluating craniocerebral injuries after a collision.

### 2.2. Finite Element Model of the Helmet

#### 2.2.1. Model Construction

**Figure 13:**
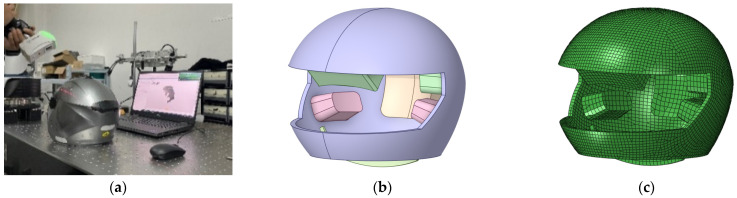
Helmet finite element model construction process: (**a**) line structured light scanning; (**b**) 3D model of the helmet; and (**c**) finite element model of helmet.

In this research project, a helmet model was created through reverse engineering. Initially, components with minimal energy absorption capacity were removed, such as the goggle rotating device, vents, cosmetic parts, and comfort liners. Only the shell, energy-absorbing liner, and strap were retained. The helmet’s fundamental structure was then scanned using a six-axis articulated arm-measuring machine equipped with a HEXAGON HP-L-8.9 T2 (Hexagon AB, Stockholm, Sweden) scanning probe (Figure 13a). The resulting point-cloud file was smoothed, denoised, and transformed into a triangular surface model. For surface creation, fitting, and thickening, the triangular surface model was imported into CATIA V5 R21 (Dassault Systèmes, Vélizy-Villacoublay, France) for geometric modeling. Finally, HyperMesh was used to mesh the imported geometric model. Figure 13b,c display the final helmet model.

**Figure 14:**
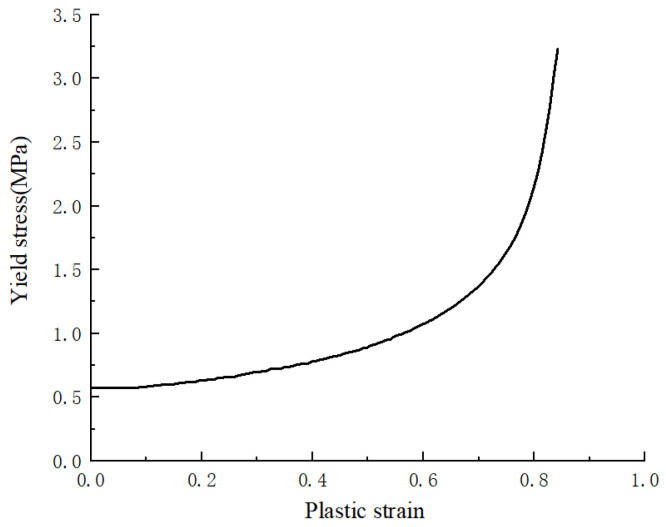
Constitutive relation (yield stress/plastic strain) of the expanded polystyrene (EPS) foam.

Table 3 enumerates the material specifications of each component of the helmet. A linear-elastic material approach was utilized to model the anvil and straps, while an elastoplastic material was adopted for the shell. A crushable foam model was used to simulate the EPS foam so as to better characterize its behavior under impact conditions. In this model, $\sigma_{Y}$ represents the von Mises stress, *k* denotes the ratio of the initial yield stress in hydrostatic compression to the yield strength in hydrostatic tension, and *k_t* represents the ratio of the initial yield stress in hydrostatic compression to the initial yield stress in uniaxial compression [36]. Figure 14 depicts how the experimental findings from Cui et al. [37] verified the stress–strain relationship of this foam, particularly for the EPS foam with a density of 80 kg m^−3^ subjected to impact loading. This ensured an accurate representation of the material’s response under impact.

#### 2.2.2. Grid Convergence

**Figure 15:**
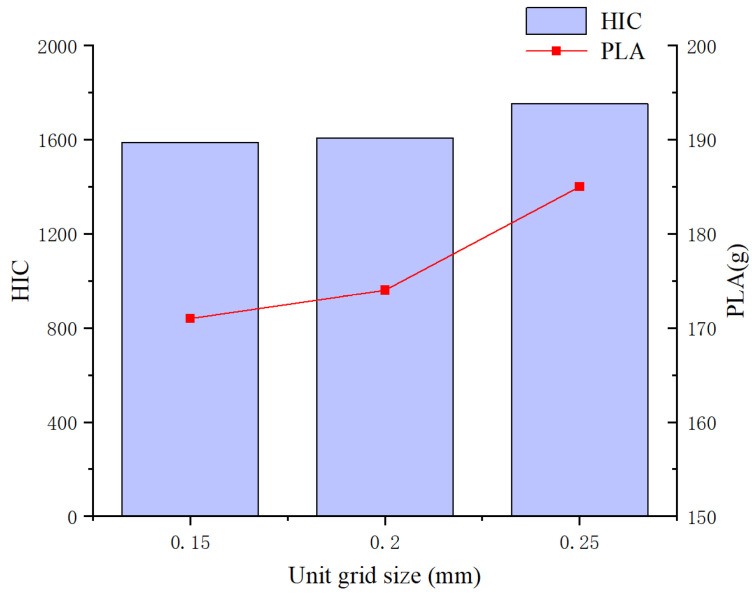
Grid convergence analysis.

To ensure the reliability of the numerical simulations, this study employed two critical injury metrics—peak linear acceleration (PLA) and head injury criterion (HIC)—as evaluation standards. The PLA and HIC values for the C2T2 configuration were analyzed across varying mesh sizes. In HyperMesh, tetrahedral meshes were categorized into the following three sizes: 0.15 mm, 0.2 mm, and 0.25 mm. The results of the finite element simulations are presented in Figure 15.

The analysis revealed that at a mesh size of 0.2 mm, both PLA and HIC values stabilized, and further mesh refinement did not significantly alter these outcomes. Balancing computational efficiency with accuracy, a mesh size of 0.2 mm was selected for the subsequent analysis of the negative-Poisson’s-ratio (NPR) structures.

#### 2.2.3. Validation of the Helmet–Head Coupling Model

**Figure 16:**
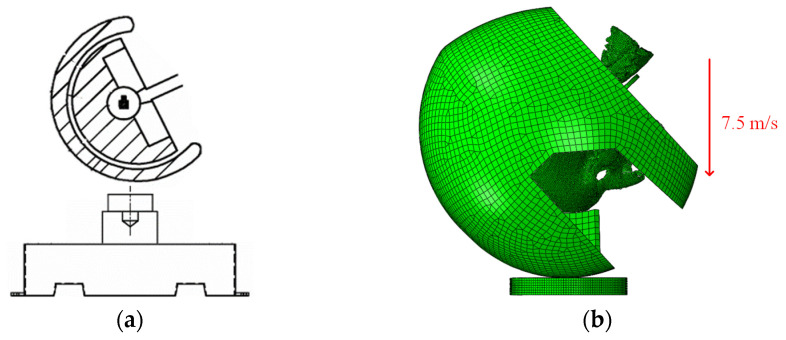
Comparison of helmet drop test: (**a**) regulation legend; and (**b**) helmet–biomechanical model.

**Figure 17:**
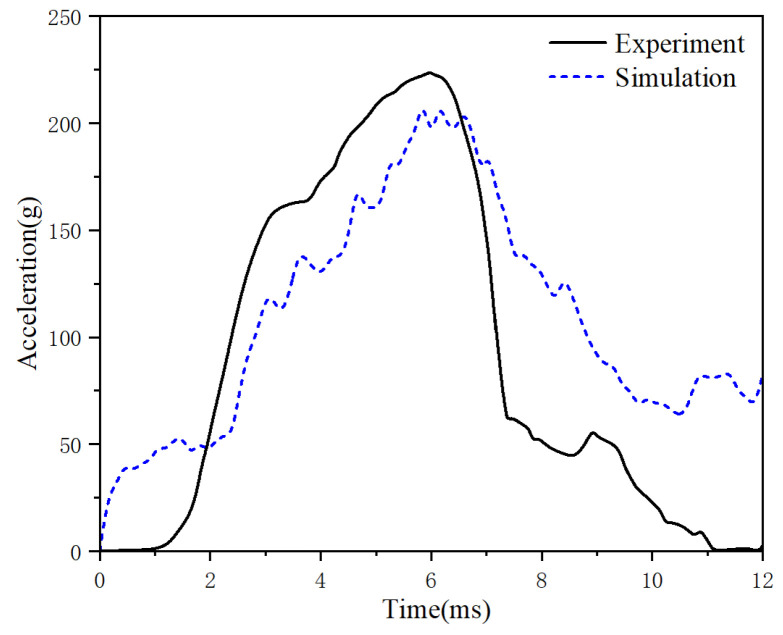
Comparison of experimental and simulated acceleration curves.

In line with the ECE R22.05 standard [13], specific helmet parts underwent drop crash tests. Figure 16 details the procedure—a robust metal head form was fixed to the M-M-model helmet and lifted 2.9 m above a flat anvil. Upon release, the helmet struck the anvil at 7.5 m s. In the simulation, the helmet–head model impacted a fixed flat anvil at the same velocity, with a −9.8 m s^−2^ acceleration applied perpendicular to the anvil’s surface to mimic gravity. Figure 17 compares the head’s center-of-mass acceleration from both tests.

Figure 17 illustrates a comparison between experimental results from the literature [39] and the simulation data of this study, showing the variation in head acceleration during the impact of the helmet on a stationary flat anvil. The figure shows that the acceleration of the helmet increases between 0 and 5 ms, corresponding to the combined acceleration of the head and helmet during the collision. As the anvil reaction force is applied, the energy-absorbing liner deforms and dissipates kinetic energy, causing the impact velocity of the head and helmet to decrease until the peak acceleration point is reached, where the velocity becomes zero. Subsequently, the helmet reaches its maximum deformation, and the head acceleration gradually decreases.

Analysis of the simulation curves and experimental data of the head’s center-of-mass acceleration reveals that the minor difference between the head model and the liner resulted in an additional collision effect during the simulation tests, leading to fluctuations and a slight lag in the peak acceleration. The numerical difference between the peak acceleration and the final acceleration was also caused by structural differences between the solid dummy head and the finite element head model. Despite these differences, the general trend and deformation process of the simulation curves generated in this study are highly similar to the experimental data in the literature [39]. This verifies the accuracy and validity of the helmet–head coupling model.

### 2.3. Negative-Poisson’s-Ratio Structural Helmet Liner Design

#### 2.3.1. Theoretical Modeling of Re-Entrant Hexagonal Honeycomb Structures

**Figure 18:**
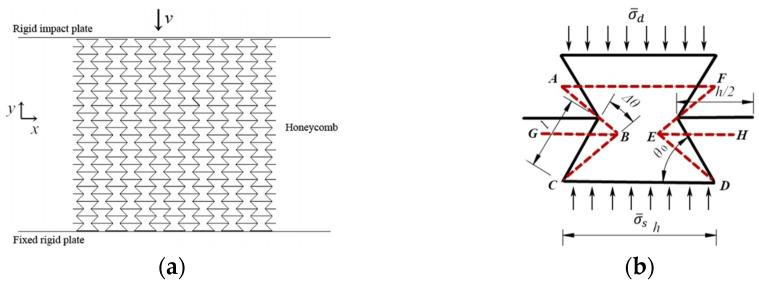
Configuration of re-entrant hexagonal honeycomb: (**a**) cellular block and (**b**) a typical re-entrant hexagonal honeycomb [42].

In light of existing research, materials of metal or polymer honeycombs are generally considered to have both elastic and fully plastic properties. As a result, in later analyses, the cell wall materials of honeycombs are assumed to have the same elastic–perfectly plastic properties. In current research on conventional hexagonal honeycombs [40,41], the cell wall material is also assumed to show elastic and fully plastic behavior. The angle between the hypotenuse and the horizontal side of the honeycomb is called the honeycomb wall angle *θ*_0_, with *h* and *l* denoting the lengths of the horizontal and hypotenuse sides of the honeycomb, respectively. To simplify the theoretical analysis, a representative honeycomb is chosen for this study. Figure 18a illustrates the compression test of a re-entrant hexagonal honeycomb block, and the structure of the deformed honeycomb is outlined by the red dashed line in Figure 18b [42].

#### 2.3.2. Poisson’s Ratio

For a representative honeycomb, as depicted in Figure 18b, when the honeycomb is subjected to compression and a change in the honeycomb wall angle (*θ*_0_) occurs due to the compression, the strains of the honeycomb in the *x*-direction and *y*-direction can be expressed as follows:

(2) $$\varepsilon_{x}=\frac{2l(cos\theta_{0}-cos(\theta_{0}-\Delta \theta ))}{2(h-lcos\theta_{0})}$$

and

(3) $$\varepsilon_{y}=\frac{2l(sin(\theta_{0}-\Delta \theta )-sin\theta_{0})}{2lsin\theta_{0}-4t},$$

Respectively, where *t* represents the thickness of the honeycomb wall. This yields the Poisson’s ratio of a representative honeycomb, as follows:

(4) $$\nu =-\frac{\varepsilon_{x}}{\varepsilon_{y}}=\frac{\sin\theta_{0}-\frac{2t}{l}}{\frac{h}{l}-\cos\theta_{0}}\cdot \frac{\cos\theta_{0}-\cos\left(\theta_{0}-\Delta \theta \right)}{\sin\theta_{0}-\sin\left(\theta_{0}-\Delta \theta \right)}$$

#### 2.3.3. Plateau Stress Under Low-Velocity Crushing

It is assumed that the cell wall length remains unchanged during cell collapse, with no bending or folding under compression. The cell wall’s kinetic energy is insignificant when loaded at low speeds or under quasi-static conditions. Therefore, only the plastic hinges at the re-entrant hexagonal honeycomb’s corners dissipate the compressive force’s work. As the honeycomb transforms from its initial state to densification (∆*θ* = *θ*_0_), the total angular change from the plastic hinges during densification is 8*θ*_0_.

Hence, the energy dissipated by the plastic hinge during the collapse of the honeycomb is as follows:

(5) $$E_{p}=M_{p}\times 8\theta_{0}=\frac{1}{4}\sigma_{Y}bt^{2}\times 8\theta_{0},$$

where $\sigma_{Y}$ denotes the yield stress of the honeycomb wall material, *b* represents the out-of-plane thickness of the honeycomb, and $M_{P}=\frac{1}{4}\sigma_{Y}bt^{2}$ represents the fully plastic bending moment of the honeycomb wall. Conversely, the work *W* conducted by the extrusion stress acting on the cell during the collapse process is as follows:

(6) $$W={\bar{\sigma }}_{s}\times H\times S=2b{\bar{\sigma }}_{s}(-lcos\theta_{0})(2lsin\theta_{0}-4t)$$

Here, $S=2b\left(h-lcos\theta_{0}\right)$ and $H=2lsin\theta_{0}-4t$ represent the cross-sectional area and the total compressive displacement of a representative honeycomb, respectively. Meanwhile ${\bar{\sigma }}_{s}$ denotes the average compressive collapse stress of the honeycomb during low-speed extrusion collapse, which also serves as the support stress acting on the bottom of the representative honeycomb because of negligible inertial effects. Subsequently, by applying the equation $W=E_{p}$, the average compressive collapse stress of the honeycomb under low-speed extrusion was calculated as follows:

(7) $${\bar{\sigma }}_{s}=\frac{\sigma_{Y}(t/l{)}^{2}\times 8\theta_{0}}{16(h/l-cos\theta_{0})(sin\theta_{0}-2t/l)}$$

### 2.4. Modelling of Helmet–Head Coupling Under Flat Anvil Impact Conditions

To boost helmet protection under flat anvil impacts, this study creates a finite element model of a novel helmet with a re-entrant hexagonal honeycomb structure. A fan-shaped cavity, suited for the honeycomb liner, is made near the ECE R22.05 [13] standard’s B-point. To use the honeycomb’s deformation and energy-absorbing abilities while keeping the helmet’s basic structure intact, the cavity’s curvature is set at π/5 (36°), with a 75 mm width and a 24 mm height, housing honeycombs in a three-row, nine-column curved layout.

**Figure 19:**
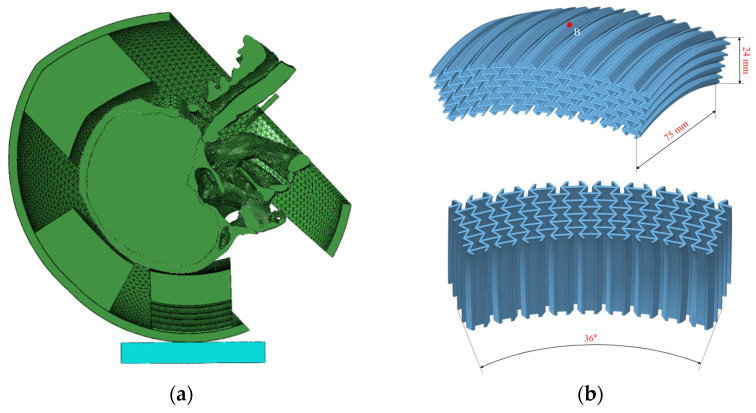
Finite element modelling of helmet with re-entrant hexagonal honeycomb structure: (**a**) section view of the coupled model and (**b**) re-entrant hexagonal honeycomb structure parameters (Point B is the collision point corresponding to the ECE R22.05 standard).

The re-entrant hexagonal honeycomb structure is curved to fit the helmet’s shape while maintaining its core structure. Given finite element simulation’s computational limits, using a full honeycomb structure in the liner would greatly lengthen the solution time. Thus, this study embeds the honeycomb only in the helmet’s forehead energy-absorbing liner layer, a key energy absorption area, replacing part of the original cushion layer, as shown in Figure 19a.

This study employs the re-entrant hexagonal honeycomb structure shown in Figure 19b, using the design parameters from Hu et al. [42] to set the final parameter combinations. The honeycomb wall angles *θ*_0_ are set at 45° and 60°, paired with wall thicknesses t of 0.8, 1.0, and 1.2 mm. These parameters are combined to produce six unique helmet liners with different characteristics, as outlined in Table 4.

## 3. Results

### 3.1. Analysis of Head Protection Performance

This study uses the head injury criterion (HIC), intracranial pressure (ICP), peak linear acceleration (PLA), and maximum principal strain (MPS) of the skull and brain to evaluate helmet protection performance [43]. It compares and analyzes the protection effectiveness of six re-entrant hexagonal honeycomb structure helmets with different parameter configurations (C1T1, C1T2, C1T3, C2T1, C2T2, and C2T3) and the original EPS helmets. The calculation of the HIC is as follows:

(8) $$HIC={\left\{(t_{2}-t_{1}){\left[\frac{1}{t_{2}-t_{1}}\int_{t_{1}}^{t_{2}}\;a(t)dt\right]}^{2.5}\right\}}_{max}$$

Here, $t_{2}-t_{1}$ represents the time interval during which the *HIC* reaches its maximum value. Typically, $t_{2}-t_{1}<15$ ms. Additionally, a(t) denotes the resultant head acceleration.

### 3.2. Head Dynamics Response

**Figure 20:**
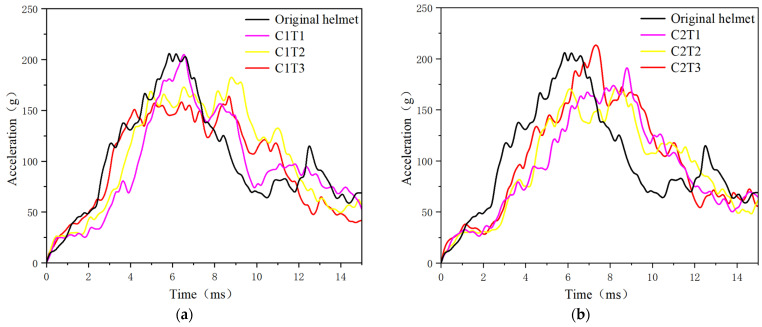
Comparison chart of head centroid acceleration: (**a**) comparison of original helmet and C1T1, C1T2, and C1T3 and (**b**) comparison of original helmet and C2T1, C2T2, and C2T3.

The protective efficacy of helmets under flat anvil impact conditions is examined by comparing the original helmet to the six re-entrant hexagonal honeycomb helmets with different parameter configurations. Figure 20 compares the head center of mass acceleration curves, whereas Table 5 compares PLA, HIC, and the PLA time point. When *θ*_0_ = 45°, C1T2 and C1T3 have lower PLA values than the original helmet. PLA continuously reduces as the honeycomb wall thickness increases. The abnormally high PLA of C1T1 is due to the wall thickness being too thin, resulting in an energy-absorbing liner structure with a large hollow area and low stability. As a result, compression collapse happens prematurely during compression, and the energy-absorbing action is ineffective, resulting in a higher PLA than the original helmet. When *θ*_0_ = 60°, the re-entrant hexagonal honeycomb helmets have lower PLA values than the original helmet in all configurations, except C2T3. Nonetheless, when the honeycomb wall thickness rises, the PLA does not decrease continuously; rather, it follows a pattern of first reducing and then increasing. The peak acceleration time and post-processing animation reveal that the PLA time point of C2T3 is 7.35 ms, which is very early. This suggests that the honeycomb structure collapses early, losing its energy-absorbing action, and the NPR effect fails to achieve the expected outcome, resulting in an anomalous increase in PLA. Regarding HIC, C1T1, C1T3, C2T1, and C2T2 have lower HIC values than the original helmets, showing that these configurations are beneficial in improving helmet protective efficacy. In contrast, C1T2 and C2T3 increase the likelihood of head injuries, which is detrimental to helmet protective efficacy enhancement.

### 3.3. Head Dynamics Response

To better illustrate each helmet’s protective effectiveness during impact compression, the study contrasts the head biomechanical reactions of the original helmet and the re-entrant hexagonal honeycomb helmets in flat anvil impact scenarios, focusing mainly on ICP and MPS.

**Figure 21:**
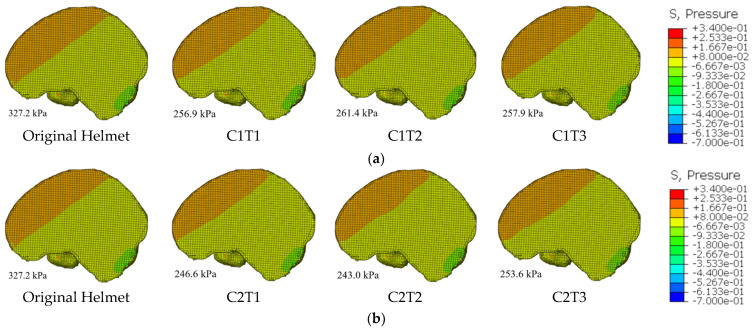
ICP cloud chart comparison: (**a**) *θ*_0_ = 45° ICP comparison cloud chart and (**b**) *θ*_0_ = 60° ICP comparison cloud chart.

Intracranial pressure is an important biomechanical metric for determining the extent of cranial injury and represents the pressure applied by substances within the cranial cavity on the skull walls. This signal is often represented by the level of cerebrospinal fluid pressure. An unusually high intracranial pressure can lead to serious complications, such as subdural hemorrhage. Figure 21 shows the cranial pressure cloud and its corresponding ICP under the flat anvil impact situation. It is clear that the original helmet’s ICP reached 327.2 kPa. According to the craniocerebral injury tolerance threshold of Tse et al. [44], an ICP exceeding 300 kPa can cause moderate brain damage. In comparison with the original helmet, all six re-entrant hexagonal honeycomb helmets with distinct parameter configurations were effective in reducing the ICP to below 300 kPa, thereby preventing moderate brain injury. Notably, C2T2 exhibited a significant ICP reduction of 25.7%, highlighting the superiority of this configuration in terms of protective performance. Additionally, when the honeycomb wall thicknesses were identical, the ICP values of the helmets with a *θ*_0_ = 60° configuration were all lower than those of the helmets with a *θ*_0_ = 45° configuration, indicating that the protective efficacy of the helmets with a *θ*_0_ = 60° configuration was more pronounced in terms of ICP protection. In terms of high-pressure zone distribution, the re-entrant hexagonal honeycomb helmet had a smaller distribution area than the original. The positive link between the ICP value and the distribution area of high-pressure zones suggests that the re-entrant hexagonal honeycomb helmet effectively reduced the distribution area of high-pressure zones.

**Figure 22:**
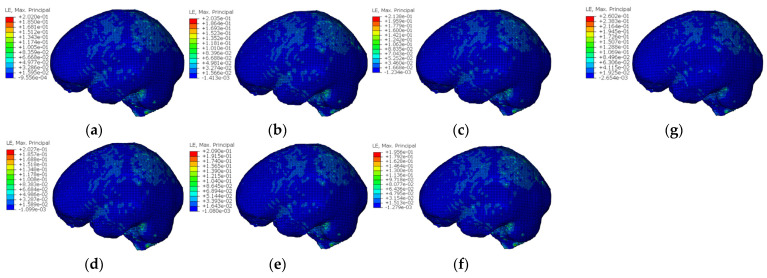
Head strain cloud chart of different parameter-configured honeycomb helmets and original helmets: (**a**) C1T1; (**b**) C1T2; (**c**) C1T3; (**d**) C2T1; (**e**) C2T2; (**f**) C2T3; and (**g**) original helmet.

The severity of craniocerebral injuries is closely linked to the mechanical responses experienced by the head during an impact. Among these responses, the maximum principal strain (MPS) is a key biomechanical parameter for evaluating brain tissue injuries. The MPS indicates the degree of localized deformation of brain tissues under an impact load, and the higher this value, the greater the risk of brain tissue injury. Figure 22 shows the cranial strain cloud for the re-entrant hexagonal honeycomb helmets with different parameter configurations compared to the original helmet. The peak MPS of the re-entrant hexagonal honeycomb helmet ranges from 0.195 to 0.213, with the region of maximum strain located at the junction of white and grey matter in the brain center. In contrast, the peak MPS of the original helmet is 0.260, with the maximum strain also occurring at this junction. The results suggest that the re-entrant hexagonal honeycomb helmet can significantly reduce the peak MPS compared to the original helmet.

Galbraith et al. [45] and Shreiber et al. [46] have reported cranial strain injury thresholds indicating that when MPS exceeds 0.20, the C1T1, C1T2, C1T3, C2T1, and C2T2 configurations may cause functional deficits in the central nervous system (CNS). In contrast, when MPS exceeds 0.25, the original helmet configurations risk causing more severe structural damage to the CNS. Notably, C2T3, with an MPS below 0.25, would only result in less severe blood–brain barrier damage to the CNS. This further highlights the superior protective performance of the re-entrant hexagonal honeycomb helmet.

**Figure 23:**
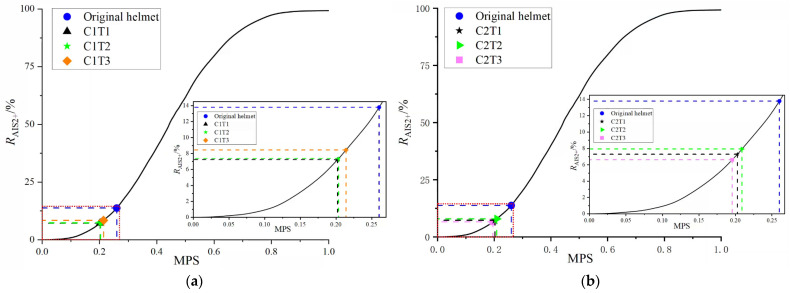
Comparison of the risk of brain injury between two kinds of honeycomb corner helmet protection: (**a**) $\theta_{0}$ = 45° and (**b**) $\theta_{0}$ = 60°.

To better illustrate the re-entrant hexagonal honeycomb helmet’s protection against brain injury, this study calculated the probability of mild brain injury risk (AIS2+), or *R*_AIS2+_, using the maximum principal strain (MPS) simulation data related to mild brain injury risk (AIS2+) [47,48]. Figure 23 shows that the original helmet had an *R*_AIS2+_ value of 13.8%. When *θ*_0_ *=* 45°, the *R*_AIS2+_ values of C1T1, C1T2, and C1T3 were all below 9%, effectively preventing brain injury. When *θ*_0_ = 60°, the *R*_AIS2+_ values of C2T1, C2T2, and C2T3 were also below 9%, significantly enhancing head protection. Notably, C2T3 had the best performance, with an *R*_AIS2+_ of 6.6%, a 7.2% reduction from the original helmet, markedly lowering brain injury risk.

## 4. Discussion

This study aimed to optimize the design of helmet energy-absorbing pads by introducing a negative-Poisson’s-ratio (NPR) concave hexagonal honeycomb structure and systematically evaluate its protective effectiveness against traumatic brain injury under flat anvil impact conditions. The results demonstrated that the helmet with a honeycomb wall angle of 60° and a wall thickness of 1.0 mm (C2T2 configuration) significantly outperformed traditional expanded polystyrene (EPS) helmets in reducing intracranial pressure (ICP) (a 25.7% decrease), peak linear acceleration (PLA), and maximum principal strain (MPS). Notably, the MPS reduction in the C2T3 configuration reached 27.7%, further lowering the probability of AIS2+ brain injury by 7.2%. Consistent with the findings of Galbraith et al. [45] and Shreiber et al. [46], the re-entrant hexagonal honeycomb helmet can mitigate injury severity to a milder blood–brain barrier injury, highlighting the unique advantages of NPR structures in optimizing energy absorption pathways. However, it is worth noting that the PLA values for C1T1 and C2T3 configurations did not show significant reductions compared to traditional EPS helmets. This can be attributed to the 0.8 mm wall thickness of C1T1, which significantly reduced the bending stiffness of the structure, leading to local buckling of thin-walled honeycomb units under impact and the premature interruption of energy absorption paths. Additionally, the smaller wall angle (45°) limited the structure’s lateral expansion capability, preventing the honeycomb units from fully achieving the expected NPR deformation mode, thereby reducing energy absorption efficiency and causing an abnormal increase in PLA. For the C2T3 configuration with a higher wall thickness (1.2 mm), although the 60° wall angle theoretically favored the NPR effect, the increased structural stiffness restricted honeycomb unit deformation during compression. This rigid deformation mode failed to effectively prolong energy absorption time, exacerbating the instantaneous impact force. As indicated by Formula 7, the average compressive stress of a honeycomb structure is proportional to the square of the wall thickness. However, an excessive wall thickness can cause deviations from ideal deformation assumptions, reducing energy absorption efficiency and potentially increasing PLA.

The elliptical annular honeycomb proposed by Zhu et al. [6] achieved a 28.03% increase in specific energy absorption (SEA) by enhancing lateral support. This study further elucidated the role of NPR structures in dispersing cranial pressure waves through dynamic impact simulations. Compared to the double-concave honeycomb studied by Hai et al. [9], the concave hexagonal honeycomb in this study demonstrated a superior efficacy in reducing ICP, likely due to its geometric configuration effectively suppressing local strain concentration. Additionally, while Qin et al. [5] reported a higher SEA for axial four chiral honeycombs under 10 m/s impacts, their study did not incorporate biomechanical parameter coupling analysis. This study addressed this gap by employing a finite element head model, clarifying the dynamic correlation between NPR structures and cranial injury indicators.

From an engineering and clinical perspective, NPR structures effectively prolong impact duration, reduce instantaneous peak forces, and thereby mitigate the risk of brain tissue injury. This has significant potential for applications in traffic safety and sports protection. Future work could integrate high-strength materials such as aluminum alloys and nylons or adaptive smart materials to further enhance structural strength and energy absorption capacity, paving the way for the development of next-generation smart helmet systems with self-healing or responsive adjustment capabilities. While this study achieved promising results through modeling and simulation, certain limitations remain. First, the NPR structure is only embedded in a partial buffer area of the helmet and cannot fully replace the EPS buffer layer. Second, the EPS foam used in this study has a limited yield strength, leading to the premature crushing of the cushion structure and an insufficient improvement in protective performance. Future experiments will explore materials such as nylon and aluminum to enhance protection. Third, this study only evaluated the helmet’s protective performance under flat anvil impact conditions. Subsequent research will incorporate additional collision scenarios, such as curbstone and circular ball anvil impacts. Finally, the cerebrospinal fluid in the finite element model was simplified as a solid, neglecting its fluid dynamics characteristics, which may affect the accuracy of stress propagation simulations. Future studies will expand impact scenarios, employ higher-strength materials, and introduce fluid–structure coupling methods to optimize model accuracy, further advancing the practical application of NPR structures in helmet design.

## 5. Conclusions

In this study, a novel helmet design method based on a negative-Poisson’s-ratio re-entrant hexagonal honeycomb liner structure is presented. The validity of the helmet–head coupling model is verified and analyzed in a crash simulation in accordance with the ECE R22.05 regulation. Through the comparison of evaluation indices such as PLA, HIC, ICP, MPS, and the probability of AIS2+ brain injury, this study explores the enhancement of the protective performance of the re-entrant hexagonal honeycomb helmet under flat anvil collision conditions. The conclusions are summarized as follows:

(1) In all the new helmet configurations, except C1T1, the peak point of head acceleration is significantly delayed compared with that of the EPS helmet. This implies that when the total impact momentum is constant, the average impact force can be reduced by prolonging the impact time, thereby decreasing the instantaneous compressive force exerted on the skull and enhancing the head protective performance.
(2) When the wall angle of the liner is increased to *θ* = 60° and the wall thickness is decreased to *t* ≤ 1.2 mm, the energy absorption efficiency of the helmet exhibits an exponential increase. The test data indicate that this configuration can reduce the HIC value from 2004 to 1549 in the baseline group.
(3) Particularly, the C2T3 helmet (wall angle 75°/wall thickness 1.2 mm) demonstrates superior head protection. It not only achieves a substantial 27.7% reduction in MPS, but also decreases the probability of AIS2+ brain injury by 7.2% through the re-configuration of the pressure wave conduction path.

The low yield strength of the EPS foam material employed in this study may constrain the energy absorption capacity of the negative-Poisson’s-ratio (NPR) structure. To address this limitation, future research will focus on exploring high-strength materials (e.g., nylon and aluminum) to enhance structural stability while carefully balancing cost and manufacturing feasibility. Additionally, the geometric parameters of the honeycomb structure will be optimized to achieve an equilibrium between protective performance and the weight and volume constraints of the helmet, ensuring both safety and practicality in real-world applications. On the other hand, the integration of NPR structures with emerging technologies such as self-healing polymers or adaptive damping systems may further revolutionize helmet design. This innovation can not only improve impact resistance, but also enable helmets to “learn” from repeated impacts and optimize their protective response over time. By bridging the gap between biomechanical protection and intelligent material innovation, the NPR based helmet showcases the future of intelligent wearable systems, where safety and functionality are synergistically developed.

## Figures and Tables

**Table 2:** Different sample collision conditions for the Trosseille experiment.

Test	Crash Site	Impactor (23.4 kg)	Impact Velocity (m s^−1^)
MS408-1	Chests	Flat Panel	5
MS408-2	Forehead	Polystyrene Foam	5
MS408-3	Face	Rigid Circles N1	5
MS428-1	Forehead	Polystyrene Foam	6
MS428-2	Face	Rigid Circles N2	7
MS428-1	Face	Rigid Circles N2	7

**Table 3:** Material parameters for helmet experiments [38].

	Element Type	Material Type	Density (kg m^−3^)	Elasticity (MPa)	Poisson’s Ratio	Yielding Strength (MPa)
Shell	Hexahedral	Linear Elastic	1150	*E* = 2000	0.37	34.3
Energy-absorbing liner	Hexahedral	Crushable Foam	80	*E* = 20; $\sigma_{Y}$ = 0.6; *k* = 1.933; *k_t* = 0.1	0.01	—
Straps	Tetrahedral	Linear Elastic	1100	*E* = 3000	0.42	—
Anvil	Hexahedral	Linear Elastic	7800	*E* = 210,000	0.3	—

**Table 4:** Material parameters for helmet experiments.

Number	Honeycomb Wall Angle ($\theta_{0}$)	Honeycomb Wall Thickness (*t*)
C1T1	45°	0.8 mm
C1T2	45°	1.0 mm
C1T3	45°	1.2 mm
C2T1	60°	0.8 mm
C2T2	60°	1.0 mm
C2T3	60°	1.2 mm

**Table 5:** Head kinematic parameters statistics.

	PLA (g)	HIC	Time Point of PLA (ms)
Original Helmet	206	1756	6.60
C1T1	207	1579	6.60
C1T2	182	2058	8.85
C1T3	171	1733	8.70
C2T1	193	1549	8.85
C2T2	174	1606	7.68
C2T3	214	2004	7.35

## Data Availability

The original contributions presented in this study are included in the article. Further inquiries can be directed to the corresponding authors.

## References

[1] China Road Traffic Accident Statistics Annual Report. https://data.stats.gov.cn/easyquery.htm?cn=C01&zb=A0S0D01&sj=2023.

[2] Li X., Li Z., Guo Z., Mo Z., Li J. (2023). A novel star-shaped honeycomb with enhanced energy absorption. Compos. Struct..

[3] Lin H.B., Liu H.T., An M.R. (2022). In-plane dynamic impact behaviors of a self-similar concentric star honeycomb with negative Poisson’s ratio. Mater. Today Commun..

[4] Song Y., Jiang S., Zhang B., Liu H., Liu F. (2025). In-Plane Impact Performance of a Gradient Sinusoidal Negative Poisson-Ratio Honeycomb Structure. J. Vib. Eng. Technol..

[5] Qin S., Deng X., Yang F., Lu Q. (2023). Energy absorption characteristics and negative Poisson’s ratio effect of axisymmetric tetrachiral honeycombs under in-plane impact. Compos. Struct..

[6] Zhu D., Wei Y., Shen X., Yan K., Yuan M., Qi S. (2024). A novel elliptical annular re-entrant auxetic honeycomb with enhanced stiffness. In. J. Mech. Sci..

[7] Yu S., Liu Z., Cao X., Liu J., Huang W., Wang Y. (2023). The compressive responses and failure behaviors of composite graded auxetic re-entrant honeycomb structure. Thin Wall Struct..

[8] Yang K., Teng F., Wang Y.B., Hu Y.H. (2023). Study on impact energy absorption performance and optimization of negative Poisson’s ratio structure. J. Braz. Soc. Mech. Sci..

[9] Hai H., Chen C., Wang W., Xu W. (2024). Impact resistance of a double re-entrant negative poisson’s ratio honeycomb structure. Phys. Scr..

[10] Wang J., Wu Z., Xiao R., Tang C., Yang Xu Y. (2023). A 3D re-entrant structural metamaterial with negative Poisson’s ratio reinforced by adding arrow structures. Smart Mater. Struct..

[11] Feng J., Liang Q., Dou Y., He J., Wu Y., Chen T. (2020). Higher stiffness hierarchical embedded strengthening honeycomb metastructure with small negative Poisson’s ratio reduction. Thin-Walled Struct..

[12] Chen H., Li F. (2023). Design, simulation and experimental verification of novel 3D metamaterial structures with negative Poisson’s ratio. Mech. Adv. Mater. Struct..

[13] (2002). Uniform Provision Concerning the Approval of Protective Helmets and Their Visors for Driver and Passengers of Motor Cycles and Mopeds.

[14] Zhou Z., Li X., Kleiven S. (2019). Fluid–structure interaction simulation of the brain–skull interface for acute subdural haematoma prediction. Biomech. Model. Mechanobiol..

[15] Willinger R., Baumgartner D. (2003). Human head tolerance limits to specific injury mechanisms. Int. J. Crashworthiness.

[16] Gao F., Yang B., Wu J., Li X., Tse K.M. (2024). Parameter optimization design of the helmet liner structure for mitigating traumatic brain injury under impact loading. Smart Mater. Struct..

[17] Willinger R., Kang H.S., Diaw B. (1999). Three-dimensional human head finite-element model validation against two experimental impacts. Ann. Biomed. Eng..

[18] Turquier F., Kang H.S., Trosseille X., Willinger R., Lavaste F., Tarriere C., Dömont A. (1996). Validation Study of a 3D Finite Element Head Model Against Experimental Data.

[19] Stalnaker R.L. (1969). Mechanical Properties of the Head. Ph.D. Thesis.

[20] Shuck L.Z., Advani S.H. (1972). Rheological response of human brain tissue in shear. ASME J. Basic Eng..

[21] Yoganandan N., Li J., Zhang J., Pintar F.A. (2009). Role of falx on brain stress-strain responses. Mechanosensitivity of the Nervous System: Forewords by Nektarios Tavernarakis and Pontus Persson.

[22] Horgan T.J., Gilchrist M.D. (2004). Influence of FE model variability in predicting brain motion and intracranial pressure changes in head impact simulations. Int. J. Crashworthiness.

[23] Tse K.M. (2013). Development of a Realistic Finite Element Model of Human Head and Its Applications to Head Injuries. Ph.D. Thesis.

[24] Tse K.M., Tan L.B., Lee S.J., Lim S.P., Lee H.P. (2014). Development and validation of two subject-specific finite element models of human head against three cadaveric experiments. Int. J. Numer. Methods Biomed..

[25] Al-Bsharat A.S. (2000). Computational Analysis of Brain Injury. Ph.D. Thesis.

[26] Westreich R.W., Courtland H.W., Nasser P., Jepsen K., Lawson W. (2007). Defining nasal cartilage elasticity: Biomechanical testing of the tripod theory based on a cantilevered model. Arch. Facial Plast. Surg..

[27] Grellmann W., Berghaus A., Haberland E.J., Jamali Y., Holweg K., Reincke K., Bierögel C. (2006). Determination of strength and deformation behavior of human cartilage for the definition of significant parameters. J. Biomed. Mater. Res. A.

[28] Kleiven S. (2006). Evaluation of head injury criteria using a finite element model validated against experiments on localized brain motion, intracerebral acceleration, and intracranial pressure. Int. J. Crashworthiness.

[29] Tanne K., Hiraga J., Kakiuchi K., Yamagata Y., Sakuda M. (1989). Biomechanical effect of anteriorly directed extraoral forces on the craniofacial complex: A study using the finite element method. Am. J. Orthod. Dentofac..

[30] Payan Y., Bettega G., Raphaël B. (1998). A biomechanical model of the human tongue and its clinical implications. Medical Image Computing and Computer-Assisted Intervention.

[31] Nahum A.M., Smith R., Ward C.C. (1977). Intracranial Pressure Dynamics During Head Impact.

[32] Trosseille X., Tarriere C., Lavaste F., Guillon F., Domont A. (1992). Development of a FEM of the Human Head According to a Specific Test Protocol.

[33] Bierbrauer J. (1996). Construction of orthogonal arrays. J. Stat. Plan. Infer..

[34] DeCock D., Stufken J. (2000). On finding mixed orthogonal arrays of strength 2 with many 2-level factors. Stat. Probab. Lett..

[35] Hardy W.N., Foster C.D., Mason M.J., Yang K.H., King A.I., Tashman S. (2001). Investigation of Head Injury Mechanisms Using Neutral Density Technology and High-Speed Biplanar X-Ray.

[36] Sandberg M., Tse K.M., Tan L.B., Lee H.P. (2018). A computational study of the EN 1078 impact test for bicycle helmets using a realistic subject-specific finite element head model. Comput. Method Biomech..

[37] Cui L., Rueda M.A.F., Gilchrist M.D. (2009). Optimisation of energy absorbing liner for equestrian helmets. Part II: Functionally graded foam liner. Mater. Design..

[38] Fahlstedt M., Halldin P., Kleiven S. (2016). The protective effect of a helmet in three bicycle accidents—A finite element study. Accid. Anal. Prev..

[39] Zheng G., Zhang X., Li S., Pang T., Li Q., Sun G. (2022). Correlation between kinematics and biomechanics of helmeted head under different impact conditions. Compos. Struct..

[40] Hu L.L., Yu T.X. (2009). Dynamic crushing strength of hexagonal honeycombs. Int. J. Impact Eng..

[41] Hu L.L., Yu T.X. (2013). Mechanical behavior of hexagonal honeycombs under low-velocity impact–theory and simulations. Int. J. Solids Struct..

[42] Hu L.L., Zhou M.Z., Deng H. (2018). Dynamic crushing response of auxetic honeycombs under large deformation: Theoretical analysis and numerical simulation. Thin-Wall Struct..

[43] Zhang X. (2022). Damage Response and Prediction of Helmet Wearing Head Under Different Impact Conditions. Master’s Thesis.

[44] Tse K.M., Lim S.P., Tan V.B.C., Lee H.P. (2014). A review of head injury and finite element head models. Am. J. Eng. Technol. Soc..

[45] Galbraith J.A., Thibault L.E., Matteson D.R. (1993). Mechanical and electrical responses of the squid giant axon to simple elongation. J. Biomech. Eng..

[46] Shreiber D.I., Bain A.C., Meaney D.F. (1997). In Vivo Thresholds for Mechanical Injury to the Blood-Brain Barrier.

[47] Wang J., Wang R., Gao W., Chen S., Wang C. (2020). Numerical investigation of impact injury of a human head during contact interaction with a windshield glazing considering mechanical failure. Int. J. Impact Eng..

[48] Meng H.S., Mao Z.Y., Ma H.X., Wang F., Xiao Z., Li G.B. (2022). Head injury risk analysis of two wheeled cyclists under the protection of airbag helmet. J. Med. Biomech..

